# Evaluating the Effectiveness of an Intervention Integrating Technology and In-Person Sexual Health Education for Adolescents (In the Know): Protocol for a Cluster Randomized Controlled Trial

**DOI:** 10.2196/18060

**Published:** 2020-08-07

**Authors:** Martha J. Decker, Abigail Gutmann-Gonzalez, Melisa Price, Julio Romero, Bhupendra Sheoran, Jennifer Yarger

**Affiliations:** 1 Department of Epidemiology and Biostatistics Philip R. Lee Institute for Health Policy Studies University of California, San Francisco San Francisco, CA United States; 2 Philip R Lee Institute for Health Policy Studies University of California San Francisco San Francisco, CA United States; 3 Fresno Economic Opportunities Commission Fresno, CA United States; 4 Youth Tech Health ETR Oakland, CA United States; 5 Philip R Lee Institute for Health Policy Studies Bixby Center for Global Reproductive Health University of California, San Francisco San Francisco, CA United States

**Keywords:** adolescents, sexual health, sex education, technology, randomized controlled trial, United States, mobile app, mobile phone

## Abstract

**Background:**

Access to a smartphone is nearly universal among American adolescents, and most of them have used the internet to seek health information. Integrating digital technologies into health program delivery may expand opportunities for youth to receive important health information, yet there are few rigorous studies assessing the effectiveness of this type of intervention.

**Objective:**

The purpose of this study is to assess the effectiveness of *In the Know* (ITK), a program integrating in-person and technology-based sexual health education for underserved adolescents.

**Methods:**

Youth were engaged in the development of the intervention, including the design of the digital technology and the curriculum content. The intervention focuses on 3 main areas: sexual health and contraceptive use, healthy relationships, and educational and career success. It includes an in-person, classroom component, along with a web-based component to complement and reinforce key content. A cluster randomized controlled trial is in progress among adolescents aged 13-19 years living in Fresno County, California. It is designed to examine the differences in self-reported health and behavioral outcomes among youth in the intervention and control groups at 3 and 9 months. Primary outcomes are condom and contraceptive use or no sex in the past 3 months and use of any clinical health services in the past 3 months. Secondary outcomes include the number of sexual partners in the past 3 months and knowledge of local clinical sexual health services. We will use mixed-effects linear and logistic regression models to assess differences between the intervention and control groups.

**Results:**

Trial enrollment began in October 2017 and ended in March 2020 with a total of 1260 participants. The mean age of the participants is 15.73 (SD 1.83) years, and 69.98% (867/1239) of the participants report being Hispanic or Latino. Study results will be available in 2021.

**Conclusions:**

ITK has the potential to improve contraceptive and clinic use among underserved youth. This trial will inform future youth-focused health interventions that are considering incorporating technology.

**International Registered Report Identifier (IRRID):**

DERR1-10.2196/18060

## Introduction

### Background

Technology-based health interventions are growing in popularity for youth. Recent promising evidence supports the feasibility and acceptability of digital interventions for knowledge and behavior change, particularly with adolescents. However, evidence of the effectiveness of this approach remains limited, with few rigorous studies assessing medium- and long-term outcomes.

#### Youth and Technology

Smartphone ownership is nearly universal in the life of American adolescents: 95% of adolescents now report either owning or having access to a smartphone, over 90% of adolescents use the internet daily, and 45% say they are online on a near-constant basis [1]. Recent statistics suggest comparable smartphone ownership among teens across gender, race and ethnicities, and socioeconomic backgrounds, with 93% of low-income adolescents reporting access to a smartphone [1]. However, research suggests key demographic differences in how youth use the internet for health. In a nationally representative survey in the United States, over 80% of adolescents reported that they had ever sought health advice on the internet, with African American and Hispanic adolescents reporting the use of web-based platforms for health information more frequently than white adolescents [2]. Lesbian, gay, bisexual, transgender, and queer (LGBTQ) youth report searching for health information online more often than heterosexual youth due to privacy-related reasons, lack of health education inclusive of their sexual orientation or gender identity, and not having anyone to ask for accurate information [3]. Integrating digital technologies into health program delivery presents an enormous opportunity to connect with youth who rely on digital health information.

Recent research assessing technology-based interventions for health has shown promise in a variety of topics and settings, including increased adherence and knowledge [4]. One systematic review of mobile apps for health and fitness found high acceptability but limited rigorous research to determine efficacy and establish evidence for best practices [5]. There is growing evidence that technology-based sexual and reproductive health (SRH) interventions can be effective [6,7]. A recent meta-analysis of 15 years of research found that technology-based interventions for youth had a significant effect on improving condom use, delaying sex, and increasing sexual health knowledge, although the effects on other sexual health outcomes were more limited [8]. Online HIV prevention interventions have shown similar efficacy as their in-person equivalents; online programs have been found to increase condom use, reduce the number of sexual partners, and reduce sexually transmitted infection (STI) incidence [9]. Similarly, Bedsider.org, a web-based educational network that provides peer feedback on birth control methods and lists youth-friendly locations to access contraceptives, has been shown to reduce unprotected sex and increase contraceptive use [10]. The enormous potential of technology-based interventions has led to the proliferation of youth-focused SRH interventions that incorporate social media, texting, and other digital components.

However, gaps remain in the research into the effectiveness of technology-based SRH interventions, particularly with certain youth populations. The analysis of long-term efficacy and the use of randomized controlled trial designs thus far are limited [8,11,12]. One systematic review of text and mobile phone app interventions for adolescents found no significant improvements in preventative sexual health behavior [13]. Furthermore, a review of apps designed for sexual health education found that the majority narrowly focused on STIs and pregnancy prevention and did not integrate evidence-based components of effective sexual health education [14]. Blended learning, which combines online and in-person instruction, is also increasing in popularity in sexual health education with mixed results [15]. Further research is needed to determine if technologies can reinforce the messaging and skill development provided in person. In addition, it is important to evaluate the viability of technology as a mechanism to reach marginalized youth populations who may not receive adequate SRH information through traditional approaches.

Sexual health education can provide critical information, but the content and quality of the curricula vary substantially [16]. Furthermore, traditional programming that focuses on pregnancy prevention often ignores the broader health and developmental issues that youth face. Incorporating educational and career success, healthy life skills, and healthy relationships into sexual health education may build youth self-efficacy in making positive life choices that impact sexual health and overall well-being [17]. However, few sexual education programs cover these more comprehensive topics [18].

#### Underserved Youth Populations

Although the adolescent pregnancy rate is declining nationwide, substantial disparities persist [19]. In addition, the rates of STIs are increasing among adolescents and disproportionately affect youth from certain racial, ethnic, geographic, and socioeconomic backgrounds [20]. Too often, sexual health education and services do not reach these youth or do not reflect their experiences and backgrounds [21]. In many cases, youth who are at the greatest need for comprehensive programming are less likely to receive it. For example, youth who frequently move, are unstably housed, or in foster care may miss school-based programming [21,22]. Similarly, few sex education curricula are designed to be inclusive of same-sex partners, sexual orientation, or gender identity [23]. In addition, many of the most common sexual health curricula were developed decades ago with limited adolescent input during the design phase. These curricula often seem dated and culturally irrelevant to youth [21].

Youth who are unstably housed, homeless, or in foster care are at a higher risk of unplanned pregnancy and STIs [24,25]. These young people often have limited access to consistent health care, are more at risk for coerced sexual activity, are more likely to have experienced trauma, and are more likely to exchange sex for money or other basic resources [24]. As a result, the rate of STI and HIV infection in homeless youth is 2 to 10 times higher than that of other adolescents [26]. Youth of color and sexual minority youth also have an elevated risk for adolescent pregnancy and STIs [19,25].

Many vulnerable youth who may need SRH services worry about provider attitudes, privacy and confidentiality, and stigma [27]. Providers are rarely competent in LGBTQ-specific health concerns, so LGBTQ youth often face discrimination when seeking health services [27]. Providing relevant information about accessible, confidential, and respectful health services for these youth can increase their use of contraceptives and improve the overall health service utilization [28].

### Objectives

The primary objective of this study is to evaluate the effectiveness of *In the Know* (ITK), an integrated in-person and technology-based sexual health education intervention, on its ability to increase contraceptive use and use of clinical health services among adolescents. The secondary objectives are to evaluate the impact of ITK on sexual risk behavior and knowledge of local clinical sexual health services as well as healthy relationship skills, career and education skills, and goal-setting skills. We hypothesize that sexually active youth who participate in the intervention will demonstrate higher contraceptive use and greater use of clinic health services as compared with youth in the control arm.

### Intervention

ITK is an innovative SRH intervention that incorporates positive youth development and a youth-centered design. ITK combines in-person sexual health education with a web-based component to provide the necessary and timely skills, information, and resources to improve the SRH and overall well-being of adolescents aged 13-19 years. In particular, it is designed to address the needs of homeless and unstably housed youth, LGBTQ youth, and youth of color.

ITK has 5 key objectives:

1. Increase use of condoms and contraceptives among those who are sexually active
2. Improve awareness about healthy relationships and decrease the incidence of sexual, physical, and emotional violence among youth
3. Improve educational and career skill development and attainment
4. Develop healthy life skills, including goal setting and stress management
5. Increase access to health care and other services through referrals and information

#### Development

Adolescents representing the diverse target populations helped to develop ITK by engaging in a user-centered design process, which recognizes that youth are the experts in their own lives [29,30]. Through a series of workshops, youth brainstormed ideas for intervention content and design and developed rapid prototypes alongside the design team. Youth then reviewed ideas and provided feedback on multiple design and content iterations.

ITK was designed using a trauma-informed approach and a positive youth development framework. This behavioral approach to adolescent development views youth as having assets that can be cultivated to reach their full developmental potential [31,32]. It focuses on creating an environment that supports protective factors, which promote personal strengths and resilience.

#### Classroom Component

The in-person, classroom component of the intervention is divided into 3 modules that can be implemented in 1 day or over the course of a few days. The total implementation time is approximately 6.5 hours, with each module lasting approximately 2 hours. *Module 1: Sexual health and contraceptive use* teaches youth the functions of the sexual and reproductive system; sexual orientation and inclusivity of all gender and sexual identities; how pregnancy occurs; birth control methods and correct condom usage; and STI prevention, symptoms, testing, and treatment options. *Module 2: Healthy relationships* helps youth understand healthy relationships, including communication, consent, and sexual violence prevention, and teaches life skills, such as stress management, identifying strengths, and goal setting. *Module 3:* *Educational and career success* informs youth of education and career options and teaches them about financial aid, resume and cover letter writing, interview skills, and budgeting.

#### Web-Based Component

**Figure 1:**
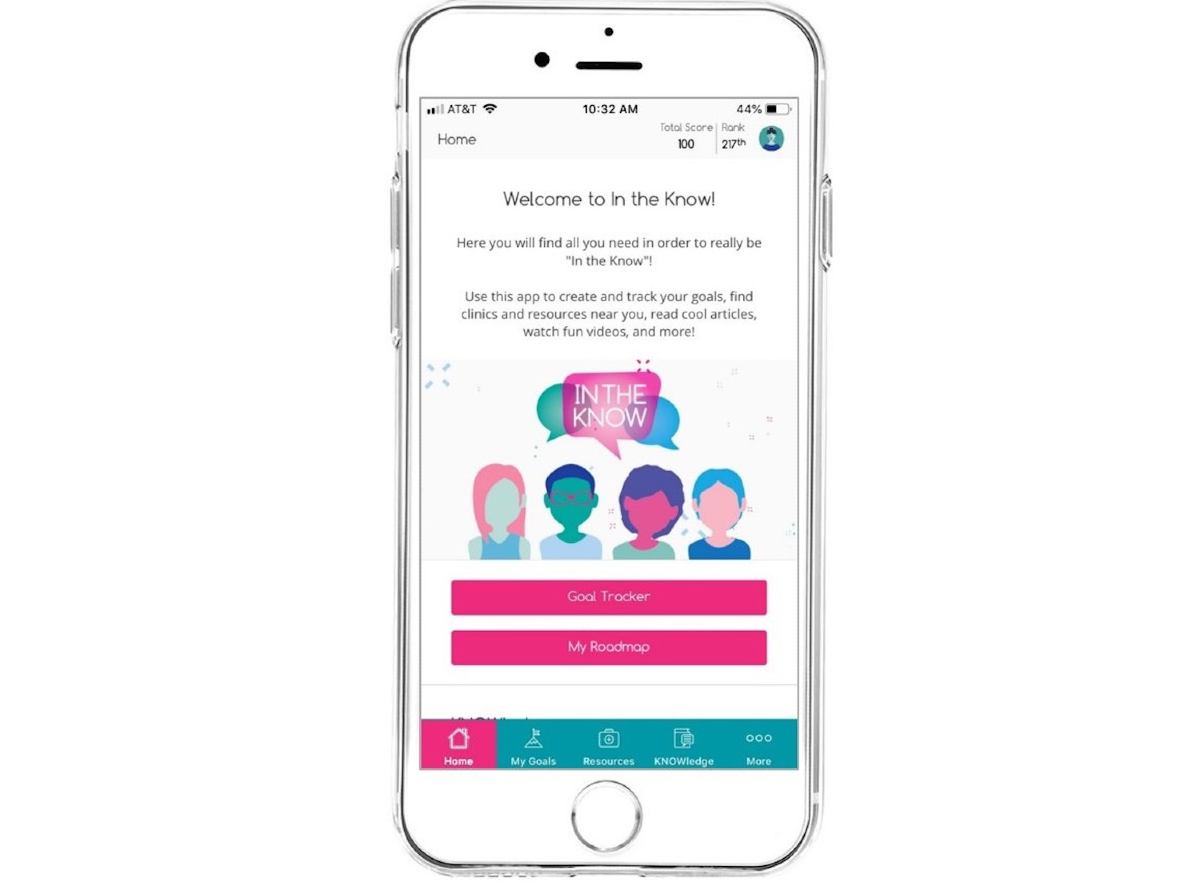
Sample screenshot from mobile application.

The web-based component incorporates digital technologies to complement and reinforce the key content covered in the classroom intervention. It uses technology-based strategies to digitalize the components of the existing curricula, including text message reminders, gamification, geo-location, and web-based resources such as videos. Youth can access this information through a downloadable app or a website (Figure 1).

This enables health educators to engage with youth on platforms easily accessed by them (such as mobile phones and tablets) to provide high-quality education and training while offering supportive tools that allow youth to practice new knowledge and skills. In addition to introducing the app and explaining how the app works at the beginning of the cohort, health educators demonstrate and support the participants in using one or more key features of the app during each session. Health educators remind participants that the app supplements the in-class education, offers reinforcement of messaging, and has additional resources that youth can access after the session. Taking the knowledge gained in the classroom, participants can generate health or career goals and set reminders to keep them on track. Participants can also complete short quizzes for points. Under the *Resources* tab, users can search for and find local services and resources using a Yelp-like feature that allows them to locate clinics on a map and rate the services after they have used them. In addition, in the *Knowledge* section of the mobile app, users can explore curated articles and videos. Youth are able to take this information and resources with them after completing the class, enabling them to refer to materials and locate services as needed. Participants also sign up to receive text messages for 1 month after the in-person sessions end, with information and reinforcement of key messages. They can also schedule reminders to complete activities to achieve their personal sexual health, relationship, and career development goals that they selected.

## Methods

This study follows the Standard Protocol Items: Recommendations for Interventional Trials (SPIRIT) guidelines [33].

### Trial Registration and Institutional Board Approval

The Institutional Review Board for the Human Research Protection Program of the University of California, San Francisco (UCSF), approved this study (IRB# 17-22381) and its protocols on September 3, 2017. This study is also registered at ClinicalTrials.gov (NCT03765255).

### Study Design

The study uses a cluster randomized controlled trial design with treatment and control groups randomized at the level of the cohort, which are defined as groups of 5 to 20 youth recruited at the same site. Researchers at the UCSF follow a simple randomization procedure. Cohorts are randomized using a computer-generated random number assignment with a 1:1 allocation. Participants in cohorts assigned to the intervention group receive ITK and those in cohorts assigned to the control group receive standard services provided at the site.

### Study Setting

The trial will be implemented by the Fresno Economic Opportunities Commission (EOC) in approximately 50 sites of youth-serving agencies in Fresno County, California. Participating agencies represent a variety of settings in which youth receive services or activities, including school and afterschool settings, employment and training sites, youth development centers, clubs, foster care sites, housing authorities, tribal agencies, and LGBTQ programs. Youth recruited in these settings generally already receive some other type of service or activity, including sports programs and clubs.

### Eligibility Criteria

Youth are eligible to participate in the study if they are aged 13-19 years, English-speaking or Spanish-speaking, and living within Fresno County at the time of enrollment.

### Recruitment

Participants will be recruited at the study sites between October 2017 and March 2020 through 3 primary mechanisms: (1) printed flyers posted at the sites, (2) EOC staff setting up a table at the site to share information about the study, and (3) staff at the participating agencies making announcements about the study.

Youth who are interested in participating in the study complete a paper-based screening form to determine their eligibility. All potential participants receive a consent form, which is available in English and Spanish. A trained member of the Fresno EOC staff reads the consent form aloud to potential participants to ensure understanding. Participants provide informed consent before completing the baseline survey.

**Figure 2:**
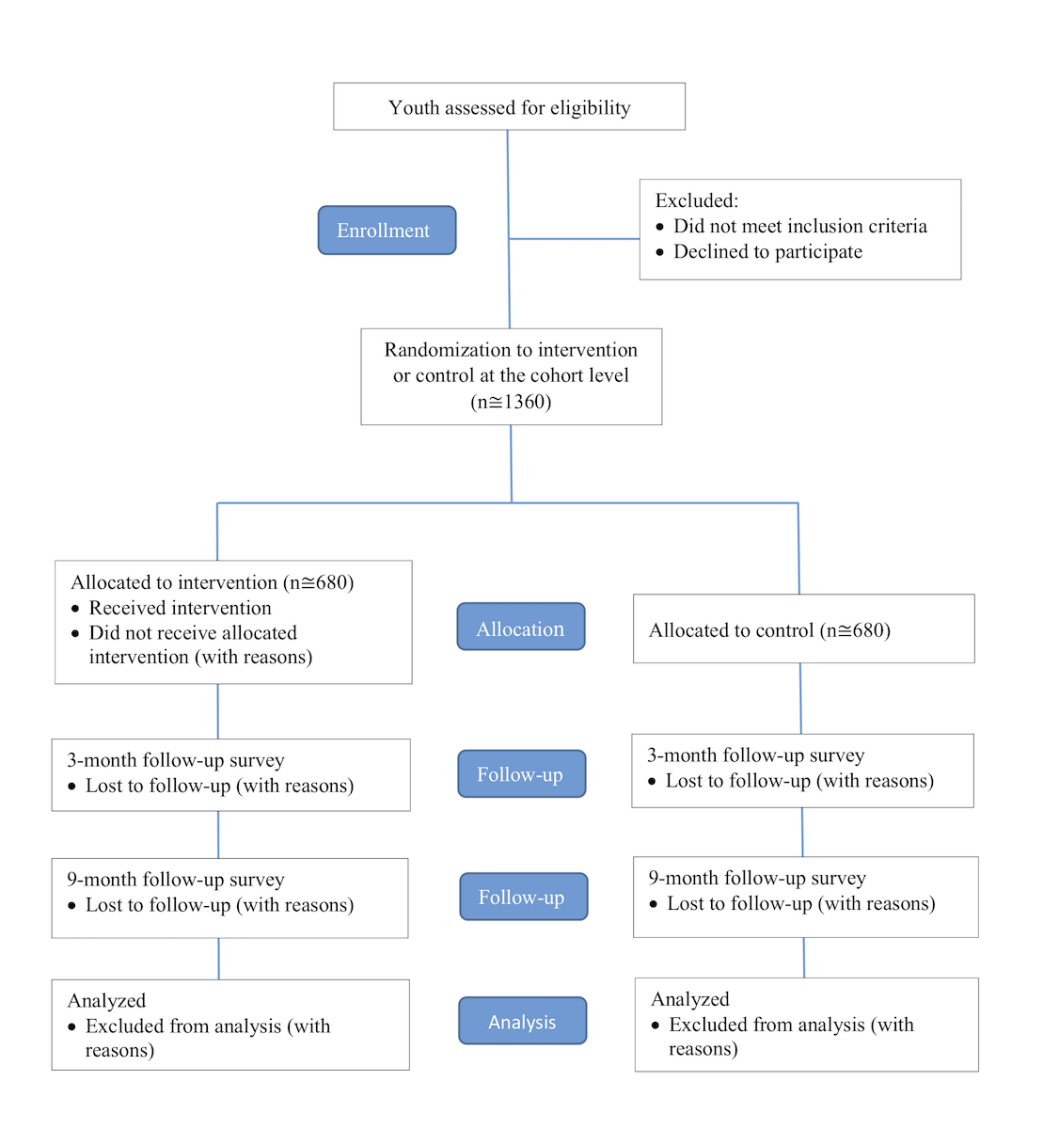
Consolidated Standards of Reporting Trials (CONSORT) flow diagram.

Each cohort’s allocation to the treatment or control group is concealed to participants and staff until enrollment and baseline data collection are complete (Figure 2). Then, a health educator opens an envelope to reveal whether the cohort has been assigned to the treatment or control group. Due to the nature of the intervention, neither the participants nor the staff can be blinded to the allocation.

### Outcomes

#### Primary Outcome Measures

The first primary outcome is condom/contraceptive use or no sex in the last 3 months. This is assessed at 3 months by asking participants how often they used birth control, including condoms, when they had vaginal sex in the past 3 months; and how often they used a condom when they had anal sex in the past 3 months. The second primary outcome is the use of any clinical health services in the last 3 months. This is assessed at 9 months by asking participants whether they have received mental health services or counseling, substance abuse treatment, or sexual health services from a doctor, counselor, therapist, social worker, or clinic in the past 3 months.

#### Secondary Outcome Measures

The secondary outcome measures are as follows:

- Number of sexual partners in the past 3 months
- Knowledge of local clinical sexual health services, which is assessed by asking “Have you heard of a clinic or doctor in your community where teens can get sexual health information and services such as condoms, birth control, pregnancy tests, STI tests/treatment, and/or HIV tests?” Response options included “yes,” “no,” and “I’m not sure.”

The outcomes and covariates are shown in Table 1. All measures are collected at baseline, 3 months, and 9 months, except where noted in the table.

**Table 1:** Outcomes and measures for In the Know.

Domains		Description
**Primary outcomes**		
	Condom/contraceptive use or no sex	Past 3 months
	Use of clinical health services	Past 3 months
**Secondary outcomes**		
	Number of sexual partners (oral, vaginal, and anal)	Past 3 months
	Knowledge of clinical sexual health services	Knows where to get sexual health information or services (yes, no, or not sure)
**Other outcomes**		
	Healthy relationship skills	Perceived ability to refuse sex and ask partner for HIV/STI^a^ testing
	Career and educational success	Current school enrollment and participation in job training or vocational program
	Goal-setting skills	Frequency of working on educational or career goal and making plans to reach goals
**Moderators and covariates**		
	Demographics	Age, race/ethnicity, and language spoken at home^b^; gender identity and sex^c^; and grade level, sexual orientation, living situation, and housing instability
	Sexual health education	Ever received and topics covered^b^
	General health education	Ever received and topics covered^b^
	Life skills education	Ever received^b^
	Technology ownership and use	Technology owned, used technology to access sexual health information and health services; location where accesses internet^b^; and websites or apps used to find health information and services^d^
	Arrest or juvenile detention history	Ever and past 3 months
	Gang-related activities	Past 3 months
	Dating violence	Past 3 months
	Cyberbullying	Ever experienced
	Sexual behavior (oral, vaginal, and anal)	Ever, frequency in the past 3 months, and drug or alcohol use before sex in the past 30 days
	Sexual and reproductive health services	Likelihood of seeking
	Tested positive for STI	Ever and past 3 months
	Pregnancy and childbearing history	Ever pregnancy and number of children
	Sexual and reproductive health knowledge	Sexual and reproductive health knowledge scale
	Communication with adults	Frequency of talking with trusted adults and comfort level talking about sex with parent
	Employment skills	Perceived skills writing a resume, cover letter, budget, or interviewing for job
	Life skills	Successfully managing stress, resolving conflict, respectful toward other, and confidently communicating ideas in the past 3 months

^a^STI: sexually transmitted infection.

^b^Measured at baseline only.

^c^Measured at baseline and 3 months.

^d^Measured at 3 and 9 months.

#### Sample Size

A target sample of 1360 youth (680 per study arm) will be enrolled in the study. The number of cohorts to be enrolled in the study is 68 per study arm, with an estimated 10 youth per cohort. We estimate 85% (1156/1360) retention at 3 months (578 per arm) and 80% (1088/1360) retention at 9 months (544 per arm).

Sample size calculations were based on the first primary (binary) outcome, which is condom/contraceptive use or no sex in the past 3 months (assessed at 3 months). We selected an intracluster correlation coefficient (ICC) of 0.02, which is within the range of previous group-randomized trials of school-based HIV, STI, and pregnancy prevention interventions in the United States [34]. With a two-sided significance level of 5% and a power of 80%, this sample size is sufficient to detect an increase in condom/contraceptive use or no sex from 60% to 67% at 3 months, with an estimated 15% loss to follow-up.

We have also planned for adequate study power for the second primary (binary) outcome, any use of clinical services in the past 3 months (at 9 months). With a two-sided significance level of 5%, a power of 80%, and an ICC of 0.02, this sample size is sufficient to detect an increase in use of clinical services from 30% to 38% at 9 months, with an estimated 20% loss to follow-up.

### Data Collection Methods

#### Baseline Survey

Participants complete the baseline survey on a tablet, although paper surveys are available as needed. The baseline survey includes questions from all domains, as shown in Table 1.

#### Follow-Up Surveys (3 and 9 Months)

All participants are asked to complete web-based follow-up surveys 3 and 9 months after baseline. A link to complete the follow-up surveys is sent via email and/or a text message to their mobile phone; participants are asked to state their preferred method of survey delivery at baseline. When the follow-up surveys are due, participants receive the survey via the method of their choice.

The 3- and 9-month follow-up surveys collect repeat measures of the outcomes measured at baseline. They also ask youth to report what websites and apps they have used to search for health information and services in the past 3 months.

#### Confidentiality

Protocols have been established to ensure the confidentiality of personal information about participants. Data are encrypted and transmitted securely through Qualtrics, a tool for collecting web-based surveys that meets the security requirements for UCSF research. Surveys are void of participant identifiers such as names and addresses. Participants are only identified through their study identification numbers. Electronic data are stored on encrypted, password-protected computers within a secure network, inside a building with limited access. Audio-recorded data, such as focus groups and interviews, and any paper-based surveys are stored in a secure and locked file cabinet. Only authorized personnel have access to the data, and all data are de-identified before analyses.

#### Retention

We expect that retention may be challenging due to the high mobility of the study’s target population. We developed a protocol to maximize retention via incentives and reminders. Study participants can receive up to US $60 for completing all surveys. The following is the incentive structure:

- US $20 for completing the baseline survey
- US $10 for completing the immediate follow-up survey (intervention only)
- US $10 for completing the 3-month follow-up survey
- US $20 for completing the 9-month follow-up survey.

At baseline, detailed contact information, including telephone numbers, email addresses, and mailing addresses, is collected. Participants are asked to provide this information for themselves as well as at least two alternative contacts, such as a parent, case manager, relative, friend, or other trusted adult. Alternate contacts are only used if the contact information for the participant is no longer accurate. EOC staff give participants small cards with the dates they will receive their follow-up surveys. Participants receive ongoing reminders about the study through text messages approximately every 2 months.

When youth are eligible to receive their follow-up survey, they are sent the survey link through their choice of text message or email. They receive two additional requests to complete the survey, at 3 and 7 days. If participants do not complete the follow-up survey during the initial outreach period, researchers call youth or their alternate contacts and offer to resend the survey or have the youth complete the survey verbally over the phone.

If the researchers are unable to contact the youth via email, text messages, or phone, EOC staff return to the implementation site, administer paper-based follow-up surveys, and collect new contact information for the youth. Surveys are mailed to the UCSF for processing.

### Statistical Methods

Our study will follow the Consolidated Standards of Reporting Trials guidelines for reporting of randomized trials, including reporting the flow of study participants through the trial (Figure 2) [35,36]. Our analysis will retain participants in their original assigned groups (intention to treat analysis) and will be conducted by a researcher who is blinded to the study arms. We will use up-to-date versions of Stata (StataCorp) to conduct all analyses. For all tests, we will use two-sided *P* values with *P*<.05 level of significance.

#### Refusal and Attrition Analysis

We will compare participants who refuse to participate and those lost to follow-up with the baseline sample to assess whether they vary by study arm, sociodemographic factors, or site. We will report any nonrandom loss to follow-up and consider the impact on the interpretation of our results.

#### Missing Data

We will report rates of and reasons for missing data, and we will assess whether participants with missing data differ systematically from others on sociodemographic characteristics, site, or trial arm. We plan to use multiple imputation to impute missing values for the predictor variables.

#### Analysis of Primary and Secondary Outcomes

The intervention arm (ITK) will be compared with the control arm (standard of care) for all primary analyses. The analysis population will include all enrolled participants. We will compare changes in condom/contraceptive use or no sex in the last 3 months from baseline to 3-month follow-up. We also will compare changes in the use of any clinical health services in the last 3 months from baseline to 9-month follow-up. We will use mixed-effects logistic regression analysis with random effects at the individual, cohort, and site levels to account for clustering. Unadjusted models will include a variable for the study group, time, and interaction between study group and time. Additional models will be estimated adjusting for sociodemographic characteristics known to be associated with the primary outcomes (age, race/ethnicity, gender, sexual orientation, rural or urban location, and housing status). We will conduct a correlation analysis among the control variables and consider the directionality of the relationship between control variables and outcome variables. For subgroup analyses, we will also test the interactions of the study group with age, race/ethnicity, gender, sexual orientation, rural or urban location, and housing status.

The analysis of the secondary outcomes will be similar to that of the primary outcomes. To compare outcomes between the treatment and control arms longitudinally, we will use mixed-effects logistic regression analyses for binary outcomes and mixed-effects linear regression for continuous outcomes.

#### Implementation Evaluation Data

In addition to the outcome data, we collect a variety of measures to verify fidelity to the curriculum, identify potential challenges, and receive feedback from youth participants. Data sources and methods include the following: immediate post survey, attendance logs, implementation logs, interviews, focus groups, site observations, and web-based analytics.

#### Immediate Post Survey

Participants in the intervention group are asked to complete a survey on the last day after all intervention activities have been completed. The immediate post survey is generally administered on tablets but is available in a paper-based format if needed. It assesses reproductive health knowledge and perceptions of the in-person and technology-based components.

#### Attendance Logs

Health educators collect attendance data for each participant. The UCSF uses this to measure the number of youth served and the amount of the intervention (dosage) they receive. The UCSF reviews attendance logs in conjunction with the number of surveys collected to ensure accuracy.

#### Implementation Logs

Conducted for each intervention cohort, this information tracks the fidelity of ITK delivery and any adaptations made. The UCSF reviews these fidelity checklists and provides technical assistance as needed.

#### Interviews

The UCSF interviews health educators annually to assess their perception of the program and identify implementation challenges and successes.

#### Focus Groups

The UCSF conducts 6-8 youth focus groups annually. A subset of participants (purposively sampled to ensure different backgrounds are represented) are invited to share their perceptions of the program and provide feedback on how it can be improved.

#### Site Observations

The UCSF conducts quarterly site observations, with sites purposively selected to represent different youth populations. They focus on factors that may affect the quality of implementation, the extent to which the intervention is delivered with fidelity, implementation challenges, and needs for additional technical assistance or training.

#### Web-Based Analytics

Web-based analytics capture the extent and type of technology used by participants, including the use of tools, resources, and referrals on the app and website at the aggregate level. This provides an important measure of dosage and utility.

## Results

This study began enrollment in October 2017, and preliminary study results will be available in 2021. As of February 2020, 1260 participants have been enrolled. Due to coronavirus (COVID-19) concerns and restrictions, cohorts scheduled for March 2020 could not be completed.

The average age of the participants is 15.73 (SD 1.83) years, and 69.98% (867/1239) of the participants report being Hispanic or Latino. When asked about their gender identity, 55.70% (694/1246) identify as female; 42.30% (527/1246) as male; 0.64% (8/1246) as transgender; and 1.36% (17/1246) as gender-queer, nonbinary, or other. In terms of sexual orientation, 81.53% (1002/1229) identify as straight and 15.79% (194/1229) identify as LGBTQ.

Study staff and their collaborating partners will share results with community members; local, state, and federal governmental officials; and other stakeholders. We will disseminate programmatic and policy implications through presentations, peer-reviewed journal articles, and social media.

The datasets generated during this study will be available from the corresponding author after completion of the study analysis on reasonable request.

## Discussion

### Strengths and Limitations

The *ITK* study has the potential to improve contraceptive and clinic use among underserved youth. Youth have been actively involved in the design and continuous improvement of the intervention, helping to ensure that the intervention is relevant and applicable to youth. Prior research suggests that the target populations’ input in the development of technology-based health interventions should be sought early in the design process to ensure short- and long-term engagement [37,38]. To our knowledge, this is one of the first longitudinal studies to examine the integration of mobile technology into a sexual health intervention, allowing us to assess program impact on youth over time. In addition to longitudinal survey data, the study will triangulate results using multiple qualitative and quantitative data sources to document implementation and provide context to the findings. A particular strength of this study is its implementation in low-income communities with youth who are often unstably housed.

This study also has some limitations. Outcomes cannot be attributed specifically to the in-person or technology component but rather are based on the combination of the two. In addition, given that the study population is highly mobile, extra measures are required to improve retention. Finally, the longitudinal study relies on self-reports of health and behavioral outcomes.

This study has faced some implementation challenges, including (1) low response rates of follow-up surveys at 3 and 9 months, particularly among early cohorts; (2) low enrollment numbers, particularly in the first months of the intervention; and (3) challenges using the mobile app technology, including internet connectivity issues, broken links within the app, youth having limited access to mobile phones, and limited mobile data plans. Successful strategies to address these challenges include diversification of follow-up strategies, such as in-person survey administration; expanded recruitment to multiple sites and scheduling cohorts 6 months in advance; and having back-up tablets and a web-based version where youth can access the app content.

### Conclusions

The results of this study can inform the development and implementation of future youth-focused health interventions that are considering incorporating technology. In addition, this study will increase the evidence regarding best practices of integrating youth-focused technology into sexual health education. Future research should compare the outcomes with populations of varying socioeconomic status and housing stability and also compare both the outcomes and cost-effectiveness of an integrated intervention with a tech-only or to an in-person–only intervention [39].

## Abbreviations

Fresno EOC: Fresno Economic Opportunities Commission
ICC: intracluster correlation coefficient
ITK: In the Know
LGBTQ: lesbian, gay, bisexual, transgender, and queer
SRH: sexual and reproductive health
STI: sexually transmitted infection
UCSF: University of California, San Francisco
